# Sucrose as an electron source for cofactor regeneration in recombinant *Escherichia coli* expressing invertase and a Baeyer Villiger monooxygenase

**DOI:** 10.1186/s12934-024-02474-2

**Published:** 2024-08-12

**Authors:** Lucija Sovic, Lenny Malihan-Yap, Gábor Szilveszter Tóth, Vilja Siitonen, Véronique Alphand, Yagut Allahverdiyeva, Robert Kourist

**Affiliations:** 1 Institute of Molecular Biotechnology, Graz University of Technology, NAWI Graz, Petersgasse 14, 8010 Graz, Austria; 2 Molecular Plant Biology, Department of Life Technologies, University of Turku, 20014 Turku, Finland; 3 Aix Marseille Univ, CNRS, Centrale Marseille, iSm2, Marseille, France; 4 ACIB GmbH, 8010 Graz, Austria

**Keywords:** Whole-cell biotransformation, Sucrose, *E. coli*, Cyanobacteria, Baeyer–Villiger monooxygenase, Oxidation, *Synechocystis* sp.

## Abstract

**Background:**

The large-scale biocatalytic application of oxidoreductases requires systems for a cost-effective and efficient regeneration of redox cofactors. These represent the major bottleneck for industrial bioproduction and an important cost factor. In this work, co-expression of the genes of invertase and a Baeyer–Villiger monooxygenase from *Burkholderia xenovorans* to *E. coli* W Δ*cscR* and *E. coli* BL21 (DE3) enabled efficient biotransformation of cyclohexanone to the polymer precursor, ε-caprolactone using sucrose as electron source for regeneration of redox cofactors, at rates comparable to glucose. *E. coli* W Δ*cscR* has a native *csc* regulon enabling sucrose utilization and is deregulated via deletion of the repressor gene (*cscR*), thus enabling sucrose uptake even at concentrations below 6 mM (2 g L^−1^). On the other hand, *E. coli* BL21 (DE3), which is widely used as an expression host does not contain a *csc* regulon.

**Results:**

Herein, we show a proof of concept where the co-expression of invertase for both *E. coli* hosts was sufficient for efficient sucrose utilization to sustain cofactor regeneration in the Baeyer–Villiger oxidation of cyclohexanone. Using *E. coli* W Δ*cscR*, a specific activity of 37 U g_DCW_^−1^ was obtained, demonstrating the suitability of the strain for recombinant gene co-expression and subsequent whole-cell biotransformation. In addition, the same co-expression cassette was transferred and investigated with *E. coli* BL21 (DE3), which showed a specific activity of 17 U g_DCW_^− 1^. Finally, biotransformation using photosynthetically-derived sucrose from *Synechocystis* S02 with *E. coli* W Δ*cscR* expressing BVMO showed complete conversion of cyclohexanone after 3 h, especially with the strain expressing the invertase gene in the periplasm.

**Conclusions:**

Results show that sucrose can be an alternative electron source to drive whole-cell biotransformations in recombinant *E. coli* strains opening novel strategies for sustainable chemical production.

**Supplementary Information:**

The online version contains supplementary material available at 10.1186/s12934-024-02474-2.

## Highlights

- Heterologous co-expression of an invertase gene improved the capacity of *E. coli* to utilize sucrose as an electron donor for whole-cell biotransformations.
- The invertase improved the efficiency of the Baeyer–Villiger oxidation of cyclohexanone catalyzed by a Baeyer–Villiger Monooxygenase both in the laboratory strain, *E. coli* BL21 (DE3) and in *E. coli* W Δ*cscR*, an *E. coli* strain bearing a deregulated sucrose operon.
- Periplasmic production of invertase for both hosts was prominent at a sucrose concentration of 10 mM.
- *E. coli* strains with invertase exported to the periplasm showed full conversion of 5 mM cyclohexanone within less than three hours using photosynthetically generated sucrose from *Synechocystis* S02.

**Supplementary Information:**

The online version contains supplementary material available at 10.1186/s12934-024-02474-2.

## Introduction

Oxidoreductases comprise a large number of enzymes catalysing oxidation and reduction reactions. Due to their high efficiency and selectivity, these enzymes find wide application in biocatalysis for pharmaceutical, fine chemical, polymer synthesis, hydrocarbon oxyfunctionalization, pollutant degradation and biosensor construction [1]. During the catalytic reaction, external compounds such as nicotinamide cofactors [NAD(P)H], ferredoxin, or adenosine triphosphate (ATP) are required in stoichiometric amounts [2]. Due to the high cost of these cofactors, their recycling by enzyme cascade reactions or through cellular metabolism is critical for economic viability [3–5]. Various chemical, electrochemical, microbial, and enzymatic methods have been developed for cofactor regeneration [2, 6]. Indeed, several metabolic engineering strategies were employed to increase NAPDH generation to improve productivity and titers in *E. coli* [7]*.* Three pathways play a major role in NADPH generation in *E. coli* namely the oxidative part of the pentose phosphate pathway, the NAD(P)^+^-dependent isocitrate dehydrogenase of the tricarboxylic acid cycle, and the transhydrogenases system [8, 9]. In whole-cell biotransformation reactions, the metabolism of microorganisms (usually heterotrophic) is used for the regeneration of redox cofactors at the expense of glucose or other carbon-rich compounds [10]. However, only a portion of the electron pairs are utilized for cofactor regeneration when glucose dehydrogenase is used [11]. In addition, the heterotrophic metabolism will necessarily dedicate a considerable part of the electrons to respiration and biomass accumulation, which results in a poor atom economy of cofactor-regeneration. Hence, cofactor regeneration using glucose results in poor atom economy [12, 13].

Interestingly, sucrose can be produced by cyanobacteria via photosynthesis which could alleviate this problem. Cyanobacteria are prokaryotes capable of utilizing CO_2_, water and basic minerals for biomass accumulation through the process of photosynthesis powered by light energy. More than 60 cyanobacterial strains accumulate sucrose as an osmoprotective compound under high salt concentrations [14, 15]. The mechanism of sucrose accumulation was exploited for the engineering of cyanobacterial strains that secrete sucrose in the range of several gram per liter. This was achieved through the expression of the sucrose regulon and by deleting competing catabolic pathways [16–18]. The feasibility of coupling microbial production strains to photosynthetic sucrose synthesis was already demonstrated in the production of L-threonine, gluconic acid and sorbitol by *E. coli, Zymomonas mobilis* and *Synechocystis* sp. PCC 6803*,* respectively [19, 20].

Cyanobacterial sucrose production presents itself as a promising option to supply electrons for the production of large-scale whole-cell biotransformations, where the low atom economy associated with the use of sacrificial electron donors is a severe problem. In this context, an industrially-relevant and important organic reaction is the Baeyer–Villiger (BV) oxidation of cyclic ketones producing lactones catalysed by oxidants such as peracids and hydrogen peroxide [21]. Due to the toxicity, instability, and lack of enantioselectivity of these catalysts, enzymatic BV oxidation has been increasingly in focus as an alternative in the last decades. Baeyer Villiger Monooxygenases (BVMOs) are flavin-dependent enzymes catalysing the oxidative transformations of ketones to their corresponding lactones and the oxidation of sulphides [22]. In the majority of BVMOs, classified as type I, molecular oxygen reacts with reduced flavin adenine nucleotide (FAD) in the active site of the enzyme; FAD is in turn regenerated by NADPH resulting in the product and water as the byproduct [23]. A kilogram-scale production of a caprolactone derivative, trimethyl-ε-caprolactone from 3,3,5-trimethyl cyclohexanone was reported by expressing a cyclohexanone monooxygenase (CHMO) from *T. municipal* in *E. coli* [24]. A higher amount of glucose relative to the final product was utilized to regenerate the cofactor contributing to higher process costs. Similarly, glycerol was also used in a kilogram-scale synthesis of a lactone relying on another CHMO [25]. Their NADPH dependency and the high relevance of the reactions they catalyse make BVMOs interesting model enzymes for the investigation of sucrose-mediated cofactor regeneration systems [26].

While cyanobacterial sucrose supply would be a suitable alternative instead of glucose for cofactor-recycling, not all industrial *E. coli* strains can metabolize sucrose. One particular strain that retained a *csc* (sucrose catabolism) regulon is *E. coli* W, which allows growth up to 2% sucrose (20 g L^−1^) [27]. By deleting the *cscR* (sucrose operon repressor) gene, *E. coli* W effectively utilized sucrose below 6 mM [28]. The efficient uptake of sucrose in *E. coli* W via active transport was made possible by a *cscB* permease, embedded in the cell membrane whereas the outer membrane is generally thought to be permeable to sucrose in *E. coli* strains and other gram-negative bacteria [29, 30]. However, solely the expression of *cscA* gene confers the capacity to grow on sucrose to *E. coli* strains which normally do not metabolize sucrose readily [31–33]. Recently, a proof of concept for whole-cell biotransformation using an *E. coli* W Δ*cscR* strain harboring a BVMO utilizing photosynthetically-produced sucrose from *Synechocystis* S02 was demonstrated [26]. Entrapment of *Synechocystis* S02 in alginate hydrogel beads resulted in ~ 9 mM (3 g L^−1^) of cumulative sucrose yield after 17 days in a semi-continuous system, which was then utilized in whole-cell biotransformation by *E. coli* W *ΔcscR* overexpressing an invertase gene with complete conversions and an average transformation rate of 0.9 mM h^−1^.

**Fig. 1:**
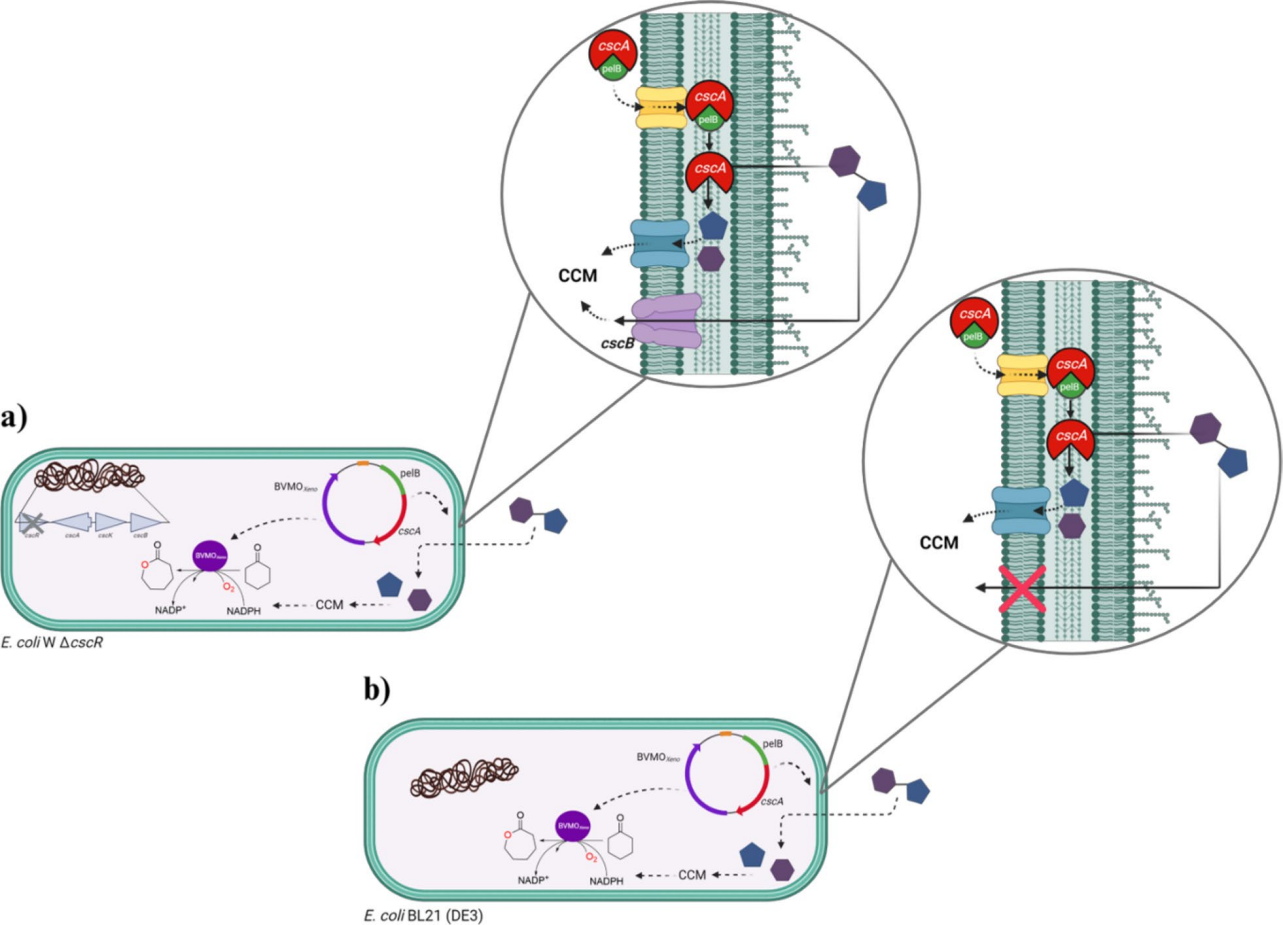
Schematic representation of sucrose utilization for cofactor regeneration in whole cell oxyfunctionalization when *cscA* hydrolyses sucrose in the periplasm. A synthetic operon was constructed containing a BVMO and a *cscA* fused with a pelB signal sequence. Both genes were controlled by a T5 promoter and cloned in a pQE-30 plasmid. The gene was heterologously expressed in a) *E. coli* W mutant with a *cscR* deletion (Δ*cscR*) and b) a standard *E. coli* strain BL21 (DE3). *E. coli* W Δ*cscR* carries the *csc* regulon encoded in the genome with a deletion of the *cscR* repressor to enable low sucrose utilization (< 6 mM) whereby *E. coli* BL21 (DE3) entirely lacks the *csc* regulon. The design of the synthetic operon can be found in Figure S1. [Abbreviations: CCM—central carbon metabolism; *cscR*—repressor, *cscA*—invertase, *cscK*—fructokinase, *cscB*—sucrose permease; pelB—signal sequence for the periplasmic export]. Image created using BioRender

While the utilization of sucrose for whole-cell redox biotransformations can be improved by the overexpression of the invertase gene, several questions remain. In particular, it is unclear whether the permease encoded in the sucrose regulon is essential, or whether simple invertase expression can boost sucrose utilization. The latter would make it easier to use laboratory strains for sucrose-driven biotransformations. The use of highly optimized laboratory strains would be particularly beneficial for difficult-to-express proteins. Herein, we report an analysis of key factors for efficient sucrose utilization of *E. coli* W Δ*cscR* strain co-expressing invertase and a BVMO with a fast reaction rate originating from *Burkholderia xenovorans* (BVMO_*Xeno*_) for the whole-cell biotransformation of cyclohexanone **1a** to the polymer precursor, ε-caprolactone **1b** (Fig. 1) [34]. Furthermore, we report that the expression of the invertase gene is a simple strategy to induce sucrose metabolism in a widely-used laboratory strain such as *E. coli* BL21 (DE3) lacking the *csc* regulon. Finally, the applicability of both systems was demonstrated in the utilization of photosynthetically-derived sucrose produced by the cyanobacterium *Synechocystis* S02 for sustainable production of platform chemicals.

## Materials and methods

### Chemicals

All chemicals were purchased from Sigma-Aldrich (Steinheim, Germany) or Carl Roth (Karlsruhe, Germany) unless otherwise indicated.

### Strain and plasmid construction

A synthetic operon containing invertase (*cscA*, accession: AF084030.1) fused with a pelB leader sequence and a BVMO gene from *Burkholderia xenovorans* (BVMO_*Xeno*_, UniProt: Q13I90) was designed and cloned into a pQE-30 vector using Gibson assembly. Both genes were controlled by a T5 bacteriophage promoter (Figure S1). As *E. coli* W does not contain a gene encoding the T7 polymerase, a T5 promoter was utilized for gene expression. This construct was used as a template for the other two cloning strategies employed in this work, i.e. (i) the construct entirely lacking *cscA* and (ii) the one carrying the *cscA* without the pelB signal sequence for the cytosolic gene expression. The vector was linearized using PCR and overhangs were introduced to the respected inserts via primers. *E. coli* TOP10 strain was used for plasmid propagation and selected mutants were screened using colony PCR with a DreamTaq Green PCR Master Mix (Thermo Scientific: K1081). Both *E. coli* W Δ*cscR* and *E. coli* BL21 (DE3) were transformed with (a) pQE-30 vector encoding BVMO_*Xeno*_::*pelB*_*cscA* cassette for the periplasmic export of *cscA*, (b) control vector expressing solely BVMO_*Xeno*_ and (c) expressing both BVMO and *cscA* into the cytosol (BVMO_*Xeno*_::*cscA*). Table 1 shows the strains and constructs utilized in this study.

**Table 1:** Bacterial strains and plasmids utilized in this study

*E. coli* Strain	Plasmid backbone pQE-30	Description	Reference
W Δ*cscR*	Wild type	*E. coli* W strain with a deleted *cscR* gene	[27]
	BVMO_*Xeno*_	BVMO gene from *Burkholderia xenovorans*	This study
	BVMO_*Xeno*_::*pelB*_*cscA*	Periplasmic expression of the synthetic gene with BVMO and *pelB*_*cscA* fusion	This study
	BVMO_*Xeno*_::*cscA*	Cytoplasmic expression of the synthetic gene with BVMO_*Xeno*_ and *cscA* fusion	This study
BL21 (DE3)	Wild type	*E. coli* B dcm ompT hsdS(rB-mB-) gal λDE3	[35]
	BVMO_*Xeno*_	BVMO gene from *Burkholderia xenovorans*	This study
	BVMO_*Xeno*_::*pelB*_*cscA*	Synthetic gene with BVMO and *pelB*_*cscA* fusion; *cscA* expressed in the periplasm	This study
	BVMO_*Xeno*_::*cscA*	Cytoplasmically expressed BVMO_*Xeno*_ and *cscA*	This study

### Bacterial strains and growth conditions on LB medium

The bacterial strains and plasmids used in this study are listed in Table S1. The *E. coli* W mutant with a *cscR* gene deletion (hereafter *E. coli* W Δ*cscR*) was provided by Professor Claudia Vickers from the University of Queensland, Australia. Seed cultures were prepared either from glycerol stocks or fresh plates and incubated overnight at 37 °C in liquid Lysogeny broth (LB) medium. Ampicillin (100 µg mL^−1^) was added to ensure selection pressure. The main culture was grown at 37 °C at 160 rpm until an optical density of 0.6–0.8 was reached.

### Growth of *E. coli* strains on different sugar sources

To test the ability of *E. coli* W Δ*cscR* and *E. coli* BL21 (DE3) to grow on sucrose and glucose, seed cultures (4 mL) were prepared in a minimal medium supplemented with 11 mM glucose. Minimal medium was prepared as follows: 100 mL of 10X M9 salts, 1 mL trace element solution, 0.05 mg mL^−1^ of thiamine, 0.05 mg mL^−1^ biotin both sterile filtered, MgSO_4_ × 7H_2_O c_final_ = 0.0081 mg mL^−1^ filled up to 1 L with sterile ddH_2_O. The main cultures were inoculated from the seed cultures to a final volume of 30 mL and OD_600_ = 0.1 using 100 mL baffled flasks. All components were autoclaved separately to avoid the precipitation of heavy salts. Glucose or sucrose (1 M prepared in ddH_2_O) was sterile-filtered before adding to the medium. The cells were left to grow at 37 °C, 120 rpm for 2 days. Control inoculum without sugar addition was also prepared in parallel.

### Enzyme production and whole-cell biotransformation of **1a**

Recombinant gene expression was induced by the addition of 1 mM isopropyl-β-D-thiogalactopyranoside (IPTG) when the cells reached an OD_600_ = 0.6–0.8 and further grown at 30 °C using the same shaking conditions. After 4–5 h of expression, the cells were centrifuged at 4 °C, 5000*g* for 30 min and washed with approximately 30 mL of Tris–HCl buffer (20 mM, pH 8.0). Once harvested, the cell suspension was centrifuged and finally resuspended in M9 salts, which were also used as a medium for whole-cell biotransformation. The cultures were adjusted to OD_600_ = 5 and pH 7.5 using NaOH in 300 mL baffled flasks (working volume = 30 mL). Sucrose (1 mM) or glucose (2 mM) was added for cofactor regeneration. The substrate **1a** (5 mM, 1 M stock solution in EtOH) was added to the cells to initiate the reaction. Control reactions were performed without sugar addition. Reactions were performed in triplicates at 25 °C and 160 rpm. Aliquots (100 µL) of the reaction mixture were taken periodically, quenched in liquid nitrogen and stored at − 20 °C before GC-FID analysis.

### Whole-cell biotransformations using sucrose produced by immobilized *Synechocystis* S02

Photosynthetically-produced sucrose from the cyanobacterium, *Synechocystis* S02 was utilized to sustain the biotransformation. The cyanobacterial cells were immobilized in alginate beads as described previously and sucrose was produced for 7 days [26]. Cells were then removed from the mixture and the sucrose-enriched BG11 medium was utilized for biotransformation. *E. coli* seed cultures were prepared overnight in LB medium supplemented with 100 µg mL^−1^ ampicillin. Main cultures were inoculated in TB medium and grown until an OD_600_ = 3 at 37 °C. After induction with 1 mM IPTG, the culture was left overnight at 25 °C for protein expression. Cells were harvested at OD_600_ = 8 and washed once with phosphate buffer and then resuspended in the sucrose-enriched BG11 medium containing 400 mM NaCl. The substrate **1a** (5 mM) and IPTG (1 mM) were added and the biotransformation was conducted at 30 °C, 1% CO_2_ and 100 rpm as previously described. Sampling was done at time points 0, 1, 2, 3 and 24 h after substrate addition and samples were analyzed on GC-FID.

### Analytics of target compounds using GC-FID and cell dry weight determination

The concentration of substrates and products was determined using Gas Chromatography with a Flame Ionization Detector (GC-FID, GC-2010 Plus Shimadzu, Japan) equipped with a ZB-5 column. Samples were extracted using 1:3 (v/v) dichloromethane containing 2 mM *n*-decanol as an internal standard. Samples were centrifuged at maximum speed for 10 min at 4 °C and the organic phase was dried using a spatula-tip of anhydrous MgSO_4_. A concentration range from 0 to 20 mM was prepared and extracted similarly to determine the concentrations for **1a** and 1b. Table S2 shows the GC-FID conditions used in this study and Figure S2 the respective calibration curves with corresponding equations. For reactions utilizing photosynthetically-produced sucrose, compounds were analysed as previously reported [26].

For cell dry weight correlations, cells were grown as described above and the enzyme was produced for up to 5 h. Afterwards, they were washed three times with sterile double distilled water (ddH_2_O) and resuspended in 30 mL LB medium to mimic biotransformation conditions. The optical density was measured and the cells were centrifuged (4 °C, 5000 × *g*) for 20 min. The supernatant was discarded and cells were placed in a 60 °C incubator and weighed until no mass difference was observed. An OD_600_ = 1 correlates to 0.3 g_DCW_ L^−1^ of cells.

### Determination of specific enzyme activity

In whole-cell biotransformations, the specific enzyme activity is given as U g_DCW_^−1^, analogous to U mg^−1^ when using a purified enzyme. Thus, it was determined based on the product formation rate (mM h^−1^), which is a slope of the curve (time *vs.* product concentration) and reflects the initial rate, i.e. ≤ 10% conversion. Since U is given in µmol min^− 1^ the conversion of the mM h^− 1^ was done and divided by 1.5 g_DCW_ L^−1^, which is the cell dry weight corresponding to the OD_600_ = 5 used to perform the whole-cell biotransformation.

## Results and discussion

### Growth of *E. coli* strains on sucrose and glucose

**Fig. 2:**
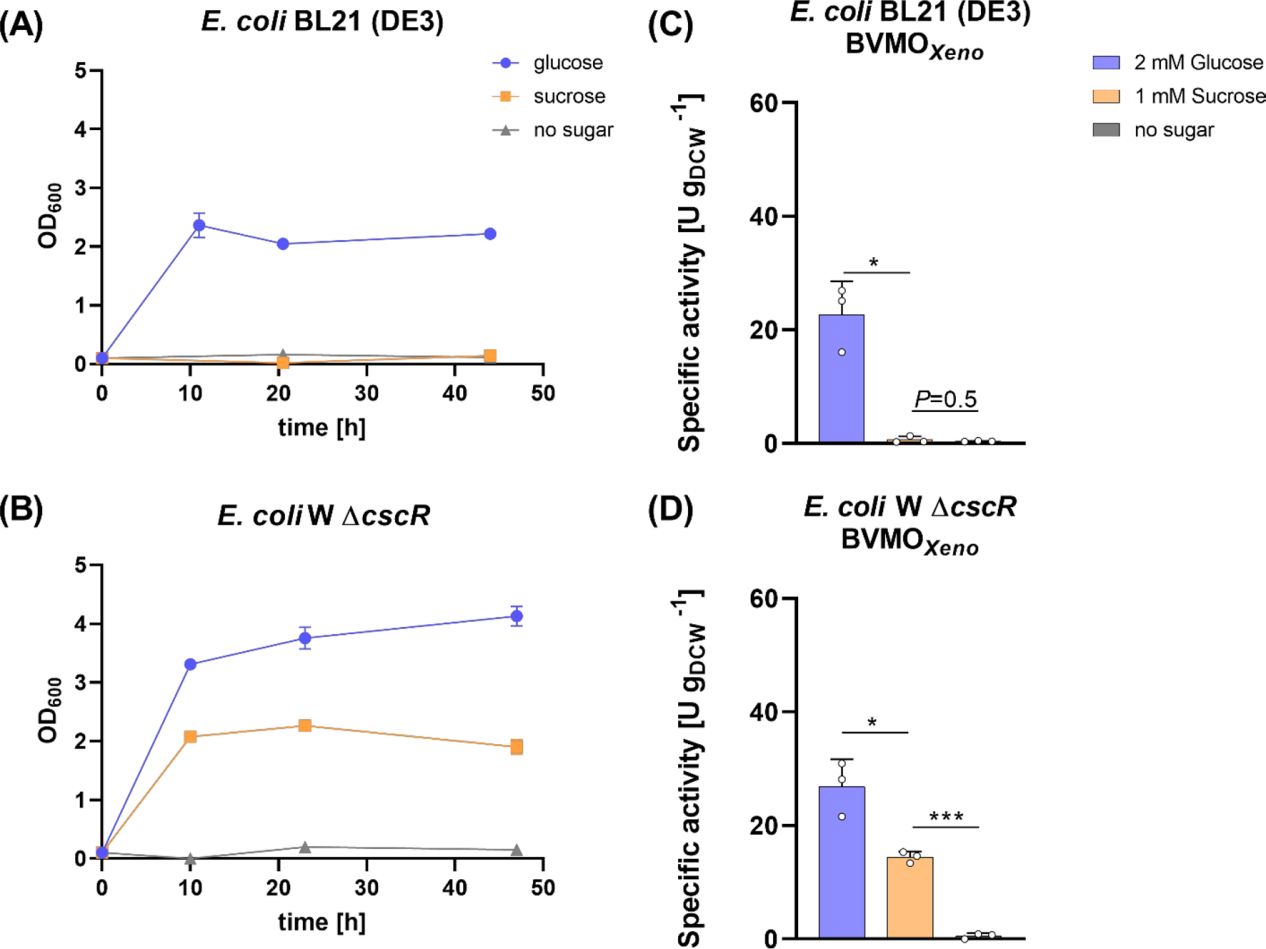
(1) Growth of (**A**) *E. coli* BL21 (DE3) and (**B**) *E. coli* W Δ*cscR* in the presence of glucose (11 mM), sucrose (6 mM) or no sugar addition in the M9 minimal medium. *Reaction conditions:* 30 mL volume in 100 mL baffled flasks, 37 °C, 120 rpm. (2) Whole-cell biotransformation of **1a** using BVMO_*Xeno*_ constructs lacking the invertase in (**C**) *E. coli* BL21 (DE3) and (**D**) *E. coli* W Δ*cscR*. *Reaction conditions:* 30 mL, M9 salts as medium, 1.5 g_DCW_ L^−1^, 5 mM 1a, 25 °C, 160 rpm, *N* = 3. All values represent data generated from three biological replicates. Error bars represent standard deviations. *P*-values were calculated using Welch’s t test (*P < 0.05)

To ascertain the metabolism of the two *E. coli* strains using the sugars tested, their growth was observed for two days in a minimal medium. In particular, we compared the sucrose utilization by *E. coli* W Δ*cscR* and *E. coli* BL21 (DE3) as a representative for frequently applied *E. coli* laboratory strains. Using native *E. coli* W, a minimum sucrose concentration of 6 mM was determined wherein catabolism takes place without activating the *cscR* gene [27, 28]. Hence, we used this concentration to monitor the growth of *E. coli* W Δ*cscR* and *E. coli* BL21 (DE3). As shown in Fig. 2, glucose was the preferred substrate for both *E. coli* strains. Unsurprisingly, *E. coli* BL21 (DE3) was not able to utilize sucrose for growth due to the lack of a *csc* regulon confirmed by a BLAST search (Figure S3) [32, 33].

Consequently, the biotransformation using sucrose in *E. coli* BL21 (DE3) (Fig. 2C) did not show any appreciable product formation comparable to negative controls. On the other hand, *E. coli* W Δ*cscR* was able to grow using sucrose albeit with a slower growth rate compared to glucose. We also observed product formation with the aforementioned *E. coli* host using sucrose to regenerate cofactors (Fig. 2D).

### Functional co-expression of invertase and BVMO in *E. coli* W Δ*cscR*

In order to analyse the utilization of sucrose in the specialized laboratory strain, we investigated the expression of the BVMO_*Xeno*_ gene and its co-expression with the invertase originating from *E. coli* W. Furthermore, we also compared the allocation of the invertase in the cytosol (*cscA*) or in the periplasm (*pelb*_*cscA*) (Table 1). Eventhough Lee and co-workers reported invertase activity [38] in the extracellular medium after intracellular expression, indicating its leakage across the cell membrane during cultivation [19], it was difficult to anticipate which system would be the most efficient for biotransformations. Successful production of BVMO_*Xeno*_ was achieved for all investigated constructs. A band at 62 kDa corresponding to BVMO_*Xeno*_ was observed in SDS-PAGE (Figure S4) [34]. However, the band for the invertase (*cscA*) at 54 kDa could not be detected, which was in agreement with previous findings from Lee et al. [19].

The strains were then investigated in the whole-cell oxidation of **1a** as shown in Fig. 1. Biotransformations were performed in the presence of either glucose (2 mM) or sucrose (1 mM) and compared with control reactions without any sugar addition. With sucrose, *E. coli* BL21 (DE3) BVMO_*Xeno*_ (the strain without invertase) did not show any product formation (Fig. 2C). In comparison, the strain had a specific activity of 23 U g_DCW_^−1^ in the presence of glucose, which shows that BVMO_*Xeno*_ was produced in soluble and active form. These results underline that *E. coli* laboratory strains without further modification do not have the capacity to utilize sucrose as also shown in Fig. 2A. We then investigated *E. coli* W Δ*cscR* BVMO_*Xeno,*_* E. coli* W Δ*cscR* BVMO_*Xeno*_*::cscA* and *E. coli* W Δ*cscR* BVMO_*Xeno*_*::pelB_cscA* to analyse whether *cscA* overexpression has an additional effect to the deregulated sucrose operon. All the constructs successfully produced the BVMO enzyme as shown in Figure S4.

*E. coli* W Δ*cscR* BVMO_*Xeno*_ showed good activity in whole-cell biotransformations driven by sucrose, indicating that the lack of the sucrose operon in BL21 (DE3) was indeed the most likely explanation for its poor sucrose utilization. The genome-encoded sucrose permease *cscB* and *cscA* invertase in *E. coli* W Δ*cscR* BVMO_*Xeno*_ appear to be responsible for the efficient utilization of sucrose as an electron source for whole-cell biotransformations [28].

**Fig. 3:**
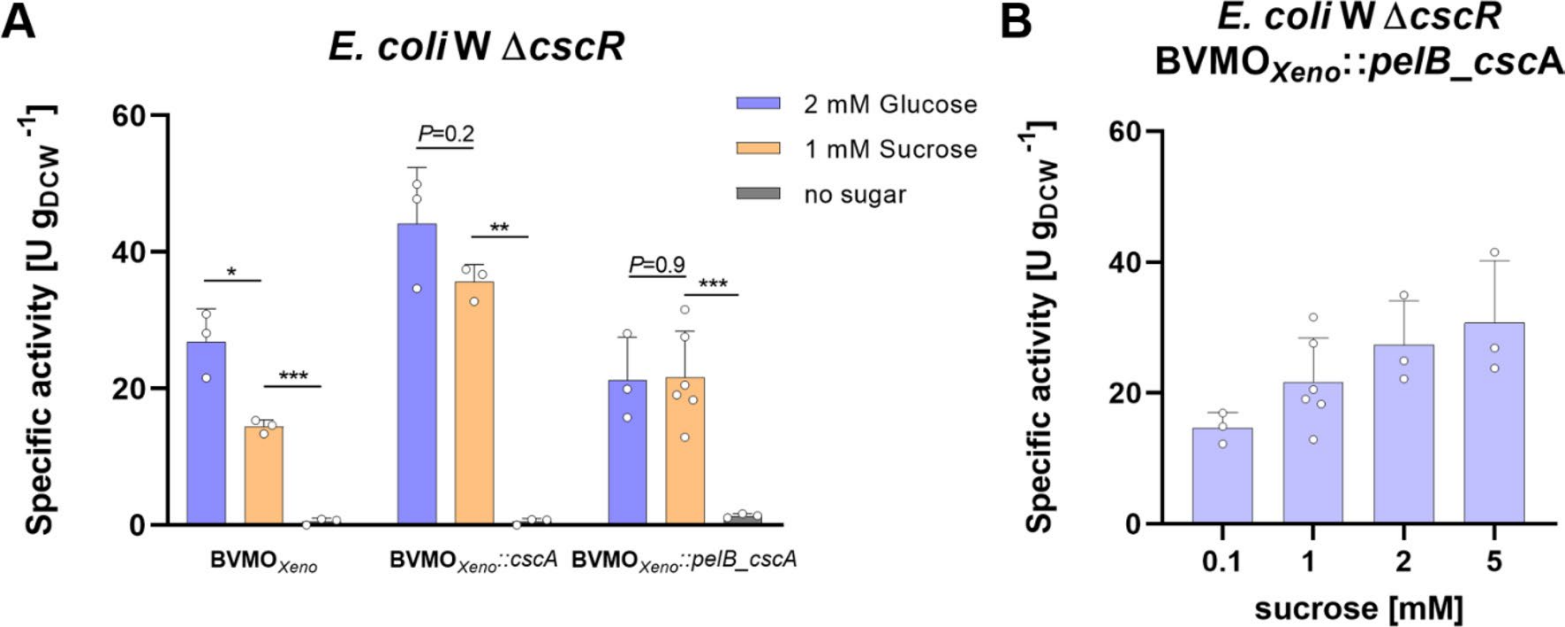
**A** Whole-cell biotransformation of **1a **mediated by different *E. coli* W Δ*cscR* constructs harboring BVMO_*Xeno*_ in the presence of various sugars and (**B**) Effect of different sucrose concentrations in the specific activity. *Reaction conditions:* 30 mL, M9 salts as medium, 1.5 g_DCW_ L^−1^, 5 mM **1a**, 25 °C, 160 rpm, *N* ≥ 3. Cofactor regeneration was ensured by adding 2 mM glucose or 1 mM sucrose. All bars represent data generated from reactions stemming from biological replicates with individual values depicted. Error bars represent standard deviations. *P* values were calculated using Welch’s t test (*P < 0.05)

Regarding the co-expression of the invertase gene in the *E. coli* W Δ*cscR* strain, both *E. coli* W Δ*cscR* BVMO_*Xeno*_*::cscA* and *E. coli* W Δ*cscR* BVMO_*Xeno*_*::pelB_cscA* showed comparable activity rates with glucose and with sucrose. The overall highest specific activity (44 U g_DCW_^−1^) was observed when glucose was added to the reaction mediated by *E. coli* W Δ*cscR* BVMO_*Xeno*_::*cscA* (Fig. 3A). This is not surprising since glucose can be easily imported by the phosphotransferase system (PTS) and is the main substrate for glycolytic pathways, including the reduction of NADP^+^ in the oxidative part of the pentose phosphate pathway, which regenerates 2 molecules of NADPH per glucose molecule [36]. Without the addition of either glucose or sucrose, only negligible product formation was observed among all constructs indicating its role in cofactor regeneration.

In the utilization of sucrose, the total activity of *E. coli* W Δ*cscR* BVMO_*Xeno*_*::cscA* of 37 U g_DCW_^−1^ was the highest of all strains investigated in this study. The higher activity of *E. coli* W Δ*cscR* BVMO_*Xeno*_*::cscA* and *E. coli* W Δ*cscR* BVMO_*Xeno*_*::pelB_cscA* indicated that overexpression of the invertase gene is also beneficial for an *E. coli* strain with deregulated sucrose operon. The specific activity did not change much with different sucrose concentrations, indicating that quite low sucrose concentrations (0.1 mM) can be utilized efficiently. With higher concentrations, there was a slight increase in activity, reaching a maximum of 31 U g_DCW_^−1^ in the presence of 5 mM sucrose. We hypothesize that with the increasing sucrose concentration, more NADP^+^ is regenerated, thus more NADPH available for the biocatalytic reaction. Consequently, the cofactor is not a limiting factor compared to when little is available within the cells. Compared to wild-type *E. coli* W that can only utilize at least 6 mM of sucrose [27, 28], the constructed *E. coli* W Δ*cscR* BVMO_*Xeno*_::*pelB_cscA* could utilize 50-fold lower sucrose concentrations (0.1 mM) delivering at least 15 U g_DCW_^−1^ of specific activity in the oxidation of **1a**.

In the presence of sucrose, *E. coli* W Δ*cscR* BVMO_*Xeno*_::*cscA* strain expressing the invertase gene in the cytosol outperformed all other constructs regardless of sugar sources (37 U g_DCW_^−1^). On the other hand, periplasmic production of invertase showed 22 U g_DCW_^− 1^. Nevertheless, both expression systems for invertase demonstrated the feasibility of utilizing low sucrose concentrations (1 mM), sustaining cofactor regeneration in an ongoing biocatalytic reaction. We hypothesized that the periplasmic expression of *cscA* would result in higher specific activities compared to the cytosolic expression because once generated in the periplasmic space, glucose, and fructose as monosaccharides can easily pass the inner membrane via the PTS system [37]. Furthermore, it would ensure the sufficient import of sugars in case *cscB* is downregulated at the investigated concentrations. The initial results at low sucrose concentrations (1 mM) did not confirm this but showed rather comparable activities between constructs with periplasmic or cytosolic gene expression. One explanation might be that when overexpressed *cscA* tends to leak out of the cells in a non-specific manner, as reported in other studies [26, 33]. Leakage of intracellularly produced *cscA* leads to sucrose hydrolysis in the extracellular medium. Its monomers glucose and fructose are then easily taken up by the cells.

### Transfer of the sucrose-utilizing ability to *E. coli* BL21 (DE3) by expression of *cscA*

**Fig. 4:**
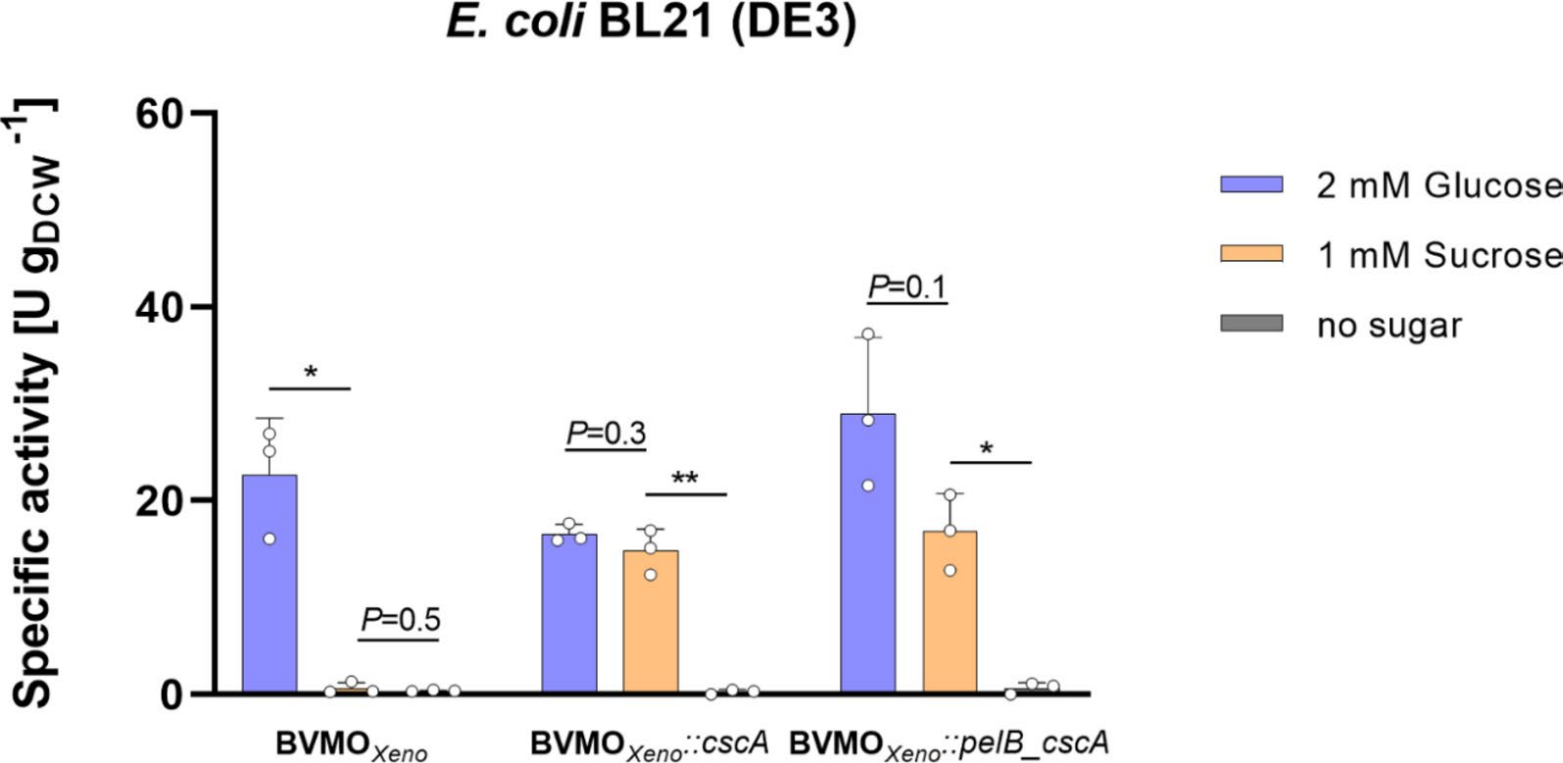
Whole-cell biotransformation of **1a **mediated by different *E. coli* BL21 (DE3) constructs harboring BVMO_*Xeno*_ in the presence of sugars (2 mM glucose or 1 mM sucrose). *Reaction conditions:* 30 mL, M9 salts as medium, 1.5 g_DCW_ L^−1^, 5 mM **1a**, 25 °C, 160 rpm, *N* ≥ 3. All bars represent data generated from reactions stemming from biological replicates with individual values depicted. Error bars represent standard deviations. *P* values were calculated using Welch’s t test (*P < 0.05)

The transferability of the system was tested in a widely-used laboratory strain, *E. coli* BL21 (DE3). Similarly, as in *E. coli* W Δ*cscR*, various constructs of the genes were expressed in *E. coli* BL21 (DE3) (Table 1). Figure 4 shows the specific activities using *E. coli* BL21 (DE3) in the whole-cell biotransformation of **1a** in the presence of either sucrose (1 mM) or glucose (2 mM). *E. coli* BL21 BVMO_*Xeno*_::*pelB_cscA* showed a specific activity of 17 U g_DCW_^−1^ with sucrose, which is very similar to the specific activities obtained with *E. coli* W Δ*cscR* (22 U g_DCW_^−1^). Moreover, this is comparable with rates obtained in *E. coli* BL21 (DE3) with the same enzyme albeit having a different promoter and carbon source [25].

Similar to *E. coli* W Δ*cscR*, overexpression of the *cscA* gene as a fusion protein with the pelB leader sequence for periplasmic export would allow hydrolysis of sucrose in the periplasm and thus transport via the phosphotransferase system. Heterologous production of *cscA* was sufficient for sucrose uptake even without *cscB* as previously demonstrated [18, 27, 33]. Sucrose can be thus used efficiently for biotransformations, without the need for production of a sucrose permease, which is not present in *E. coli* BL21 (DE3) constructs. As it is known that invertase is leaking to some extent out of the cells [19], this amount of invertase appears to be sufficient for the extracellular (or periplasmic) hydrolysis of sucrose. At a sucrose concentration of 1 mM, periplasmic or cytosolic production of invertase did not lead to significant differences in the specific activity (Fig. 4). In fact, there were no significant differences when the invertase gene was expressed either in the periplasm or in the cytosol for *E. coli* BL21 (DE3), indicating that, in principle, both strategies are equally applicable for biotransformation applications in laboratory strains of *E. coli* BL21 (DE3). We postulated that the outer membrane of *E. coli* cells is permeable for sucrose and its insertion in the cell could be limited by the inner membrane. However, *cscA* again showed to have the ability to confer sucrose uptake without significant difference whether produced in the periplasm or in the cytosol. Thus, sucrose can be used as an alternative electron source for BVMO oxidation of **1a** in the laboratory strain *E. coli* BL21 (DE3), simply by co-expressing the heterologous *cscA* gene.

### Photosynthetically-derived sucrose fuels oxidation reaction using recombinant *E. coli*

**Fig. 5:**
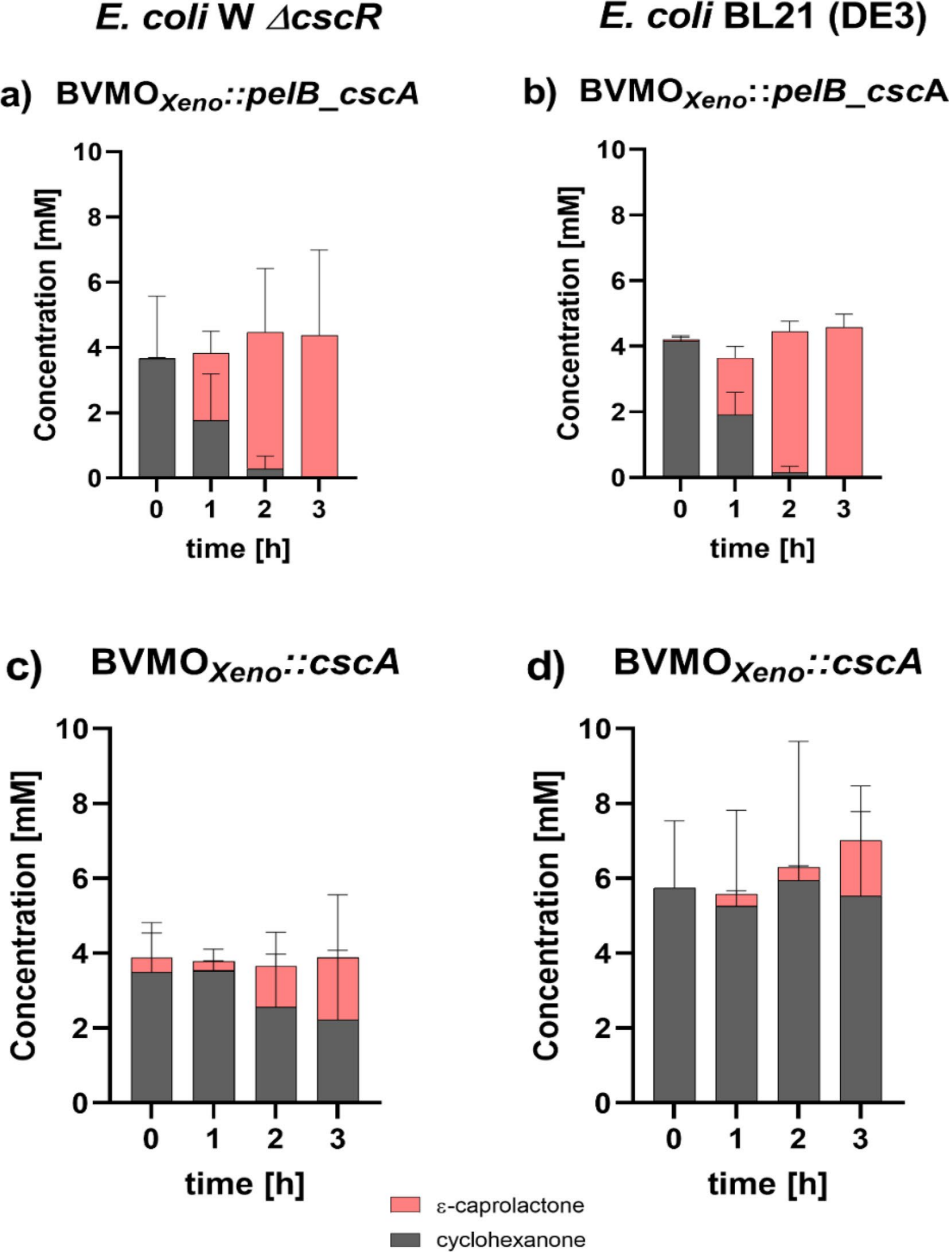
Progress of whole-cell biotransformation of **1a** mediated by BVMO_*Xeno*_::*pelB_cscA* produced in (**a**) *E. coli* W Δ*cscR* and (**b**) *E. coli* BL21 (DE3) or by BVMO_*Xeno*_::*cscA* produced in (**c**) *E. coli* W Δ*cscR* and (**d**) *E. coli* BL21 (DE3) supplemented by photosynthetically-derived sucrose from *Synechocystis* S02. *Reaction conditions:* sucrose-enriched BG11 medium containing 400 mM NaCl, 1% CO_2_, 30 °C, 100 rpm, initial concentration of 5 mM **1a**, *N* = 3. All bars represent data generated from reactions stemming from biological replicates with individual values depicted. Error bars represent standard deviations

Finally, the feasibility of utilizing photosynthetically-derived sucrose to regenerate cofactors during whole-cell biotransformation of **1a** in the constructed *E. coli* strains was demonstrated. Sucrose (*ca*. 9 mM) was produced over 7 days by *Synechocystis* S02 immobilized in alginate beads. Afterwards, *E. coli* strains using different plasmid constructs (Table 1) were inoculated in the sucrose-enriched medium and **1a** was added to initialize the reaction. Figure 5 shows the progression of the biotransformation mediated by *E. coli* W Δ*cscR* and *E. coli* BL21 (DE3) with a periplasmic and cytoplasmic transport of invertase.

Both *E. coli* strains with a periplasmic production of invertase (Fig. 5a and b) showed efficient utilization of sucrose with a complete conversion of **1a** after 3 h. In contrast, biotransformations performed using strains with cytosolic production of invertase, which requires sucrose import across the inner cell membrane, showed lower product formation and the reaction did not proceed to completion (Fig. 5c and d). Negative controls without the invertase in *E. coli* BL21 (DE3) (Figure S5) did not show any product formation. *E. coli* W Δ*cscR* BVMO_*Xeno*_::*pelB_cscA* was deemed to be the best-performing strain resulting in a volumetric productivity of 1.7 mmol L^−1^ h^−1^. Interestingly, also *E. coli* BL21 (DE3) BVMO_*Xeno*_::*pelB_cscA* achieved complete conversion within a short time, showing that the introduction of the sucrose-utilizing capacity to this laboratory strain was straightforward.

Using photosynthetically-produced sucrose, constructs expressing the invertase in the periplasm performed better as compared with cytosolic expression (Fig. 5). This was observed both for *E. coli* W Δ*cscR* and BL21 (DE) strains. Interestingly, in the experiments using pure sugars, intracellular and periplasmic production had worked equally well (Figs. 3 and 4). A possible explanation is that at the six-fold higher sucrose concentrations used in the system with photosynthetically-derived sucrose, hydrolysis of the sucrose in the periplasm is more efficient than leaking of invertase. Moreover, the leaking of invertase might differ under different conditions of biotransformation, making the periplasmic invertase production the more reliable option. Also, cell permeabilization with the effect of cell activity is not expected, this is more likely when using lyophilized cells. Resting cells, on the contrary, still require the pentose phosphate pathway and perform glycolysis.

In summary, a stronger effect of the periplasmic invertase production was observed when higher concentrations were applied using higher photosynthetically-derived sucrose when determining the initial specific activity of BVMO oxidation reaction using respective strains.

Notably, Fig. 5 shows no particular difference between the *E. coli* W Δ*cscR* and BL21 (DE3) strains while carrying the same BVMO_*Xeno*_::*cscA* cassette*,* whereas the same variants resulted in different activities in the initial study (Figs. 3 and 4). We hypothesize that when small amounts of sucrose such as 1 mM are used the genomically encoded sucrose permease *cscB* and invertase *cscA* in the *E. coli* W *ΔcscR* add to more effective import and hydrolysis, respectively. Thus, enabling the biocatalytic reaction to take place at a higher rate. As a result, more NADPH is available to the enzyme, as opposed to BL21 (DE3), which solely relies on the recombinant invertase. However, when sucrose is abundant the hydrolysis is faster, and NADP^+^ reduction is taking place at a higher rate than the BVMO_*Xeno*_ oxidation. Thus, NADPH is not the rate-limiting factor and the effect of the genomically encoded enzymes is less prominent reflecting no particular changes in the specific enzyme activity.

Additionally, results in Fig. 5 represent the quantification of a total substrate and product each hour and not the enzyme specific activity, which is determined within the *ca.* first 15 min (≤ 10% conversion) of the reaction. Consequently, the experiments involving pure sugars focus on reaction rates and directly correlate them to different sugar types and the metabolism of the respective host. On the other hand, when a given amount of photosynthetically-derived sucrose was used instead, the focus was on how long it takes for the enzyme to drive the oxidation of **1a** to completion while using the thus far fastest BVMO variant. Additionally, if different *E. coli* variants have a different effect within this time frame, which was a benchmark based on previous studies [26, 34].

The volumetric productivity of the system using photosynthetically-derived sucrose is still lower than that of the recombinant cyanobacteria (up to 3.7 mmol L^−1^ h^−1^), but the high efficiency of *E. coli* whole-cell catalysts in Baeyer–Villiger oxidation reactions of up to 1.7 mmol L^−1^ h^−1^ shows the outstanding potential of this host [34]. We believe that our present work will contribute to a coupling of this system to sustainable photosynthetic sucrose production.

### Periplasmic production of invertase is advantageous when high sucrose concentration is used

In the initial experiments, we tested for sucrose utilization at 1 mM concentration because of the assumption that high concentrations are ideal conditions. Also, sugars are costly and hence can be a bottleneck in a biotechnological process. Given that, if a batch of photosynthetically-produced sucrose results in low yields, in the case of effective *E. coli* strains, they will subsequently perform the biocatalysis at high rates. Furthermore, *E. coli* ferments sugars when highly abundant and the Crabtree effect is a possible unfavorable outcome [39].

**Fig. 6:**
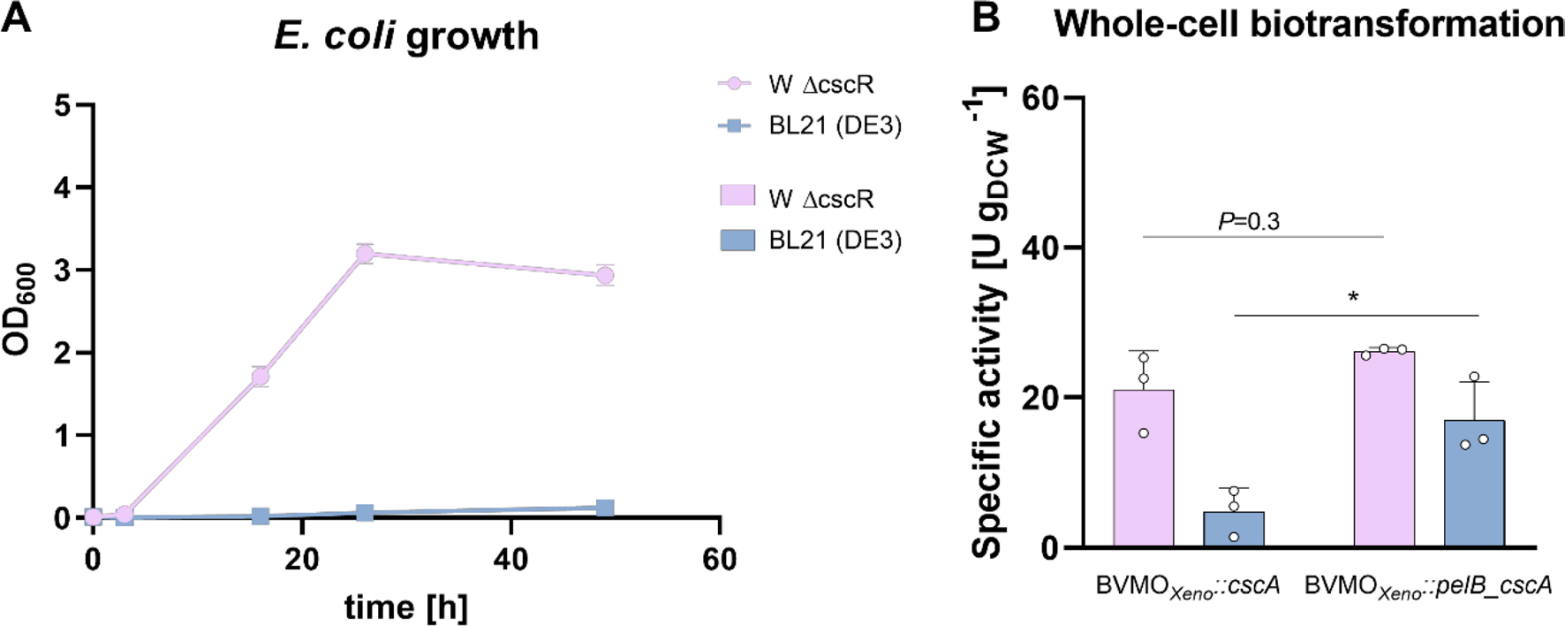
**A** Growth of *E. coli* W Δ*cscR* and *E. coli* BL21 (DE3) in the presence 10 mM sucrose in M9 minimal medium. *Reaction conditions:* 30 mL volume in 100 mL baffled flasks, 37 °C, 120 rpm; and **B** Specific enzyme activities obtained in whole-cell biotransformation of **1a** mediated by BVMO_*Xeno*_::*cscA* and BVMO_*Xeno*_::*pelB_cscA* produced in *E. coli* W Δ*cscR* and *E. coli* BL21 (DE3) when supplemented with 10 mM sucrose. *Reaction conditions:* 30 mL, M9 salts as medium, 1.5 g_DCW_ L^−1^, 5 mM **1a**, 25 °C, 160 rpm, *N* = 3. All values represent data generated from three biological replicates. Error bars represent standard deviations. *P*-values were calculated using Welch’s t test (*P < 0.05)

However, we hypothesized that the higher specific enzyme activities with photosynthetically-derived sucrose attributed to the higher sucrose availability, which was hence confirmed by the results shown in Fig. 6B referring to whole-cell biotransformation of **1a **and the addition of 10 mM pure sucrose. These indicate that periplasmic invertase production was overall the most efficient for the oxidation of **1a** when using 10 mM sucrose. *E. coli* W *ΔcscR* and BL21 (DE3) resulted in 26 U g_CDW_^−1^ and 17 U g_CDW_^−1^, respectively. Same as with the photosynthetically-derived sucrose, *E. coli* BL21 (DE3) BVMO_*Xeno*_::*cscA* was the worst performing under these conditions, and *E. coli* W Δ*cscR* BVMO_*Xeno*_::*pelB*_*cscA* the best one. In addition, Fig. 6A shows the growth of both *E. coli* W Δ*cscR* and BL21 (DE3) strains in the presence of 10 mM sucrose. Results were comparable to those in Fig. 2A and B.

These final results indicate that indeed if higher concentrations of sucrose in solution are applied to drive the oxidation of **1a**, the cofactor regeneration is faster if the recombinant invertase is produced in the periplasmic space thus overcoming a possible bottleneck of NADPH limitation.

## Conclusion

The co-expression of invertase and BVMO in two *E. coli* chassis showed efficient utilization of sucrose to fuel a whole-cell monooxygenase reaction. Coupling sucrose-utilizing strains with sucrose-secreting cyanobacteria poses the challenge that many of the frequently used and well-characterized *E. coli* laboratory strains do not have the capacity to utilize sucrose. Specific *E. coli* strains that have retained permeases and invertases for sucrose utilization are available but are less practicable for research since they lack the above-mentioned features (such as gene deletions to facilitate disulphide bond formation). Our results show that on the one hand, the introduction of the capacity of sucrose utilization into laboratory strains allows relying on the large experience with these well-characterized systems and harnessing the diversity of features available for these strains (as a ‘plug-and-play system’) whose introduction into *E. coli* W Δ*cscR* would be tedious. This is a great advantage for research purposes. On the other hand, invertase overexpression also improved the performance of *E. coli* W Δ*cscR*, showing that a combination of a deregulated sucrose operon with additional periplasmic invertase expression leads to the highest efficiency in the coupling of whole-cell biotransformations in *E. coli* with photosynthetically-derived sucrose, where alginate immobilized cyanobacteria can easily be removed – which makes this system highly attractive for sustainable, sucrose-driven production processes.

### Supplementary Information

Supplementary Material 1.

## Data Availability

All data generated or analysed during this study are included in this published article and its Additional file.

## Abbreviations

**1a**: Cyclohexanone
**1b**: ε-Caprolactone
ATP: Adenosine triphosphate
BVMO: Baeyer Villiger Monooxygenase
BVMO_*Xeno*_: BVMO from *Burkholderia xenovorans*
BVMO_*Parvi*_: BVMO from *Parvibaculum lavamentivorans*
CHMO: Cyclohexanone monooxygenase
*cscR*: Sucrose repressor gene
*cscA*: Invertase gene
*cscB*: Sucrose permease gene
*cscK*: Fructokinase gene
*E. coli*: *Escherichia coli*
*E. coli* W Δ*cscR*: *E. coli* W with a deleted sucrose repressor
FAD: Flavin adenine dinucleotide
GC-FID: Gas Chromatography equipped with a Flame Ionization Detector
IPTG: Isopropyl-β-D-thiogalactopyranoside
NAD(P)H: Nicotinamide adenine dinucleotide (phosphate)
OD_600_: Optical density at 600 nm
pelB: Signal sequence for the periplasmic export
*Synechocystis* sp.: *Synechocystis* sp. PCC 6803
