# Supplementary material for: Sucrose as an electron source for cofactor regeneration in recombinant Escherichia coli expressing invertase and a Baeyer Villiger monooxygenase
Source: Microb Cell Fact. 2024 Aug 12;23:227. doi: 10.1186/s12934-024-02474-2 (PMC11318132; doi:10.1186/s12934-024-02474-2)
Supplement: Supplementary file 1 — Supplementary Material 1. [file 12934_2024_2474_MOESM1_ESM.pdf]

Supporting Information

Sucrose as an electron source for cofactor regeneration in recombinant *Escherichia coli* expressing invertase and a Baeyer Villiger Monooxygenase

Lucija Sovic[1], Lenny Malihan-Yap[1], Gábor Szilveszter Tóth[2], Véronique Alphand[3], Vilja Siitonen[2], Yagut Allahverdiyeva[2], Robert Kourist*[1,4]

[1]Institute of Molecular Biotechnology, Graz University of Technology, NAWI Graz, Petersgasse 14, 8010 Graz, Austria

[2]Molecular Plant Biology, Department of Life Technologies, University of Turku, 20014 Turku, Finland

[3]Aix Marseille Univ, CNRS, Centrale Marseille, iSm2, France

[4]ACIB GmbH, 8010 Graz, Austria

*Correspondence: kourist@tugraz.at

Author information

Lucija Sovic	sovic@tugraz.at
Lenny Malihan-Yap	lenny.yap@tugraz.at
Gábor Szilveszter Tóth	gabor.toth@utu.fi
Vilja Siitonen	vksiit@utu.fi
Véronique Alphand	v.alphand@univ-amu.fr
Yagut Allahverdiyeva	allahve@utu.fi
Robert Kourist	kourist@tugraz.at

Cover letter

This manuscript fits the aims of the *Microbial Cell Factories* journal since it presents the development of two microbial chassis, which act as biocatalysts for producing valuable precursor molecule interesting for industrial production.

The authors declare no issues relating to journal policies and no potential competing interests.

All authors have approved the manuscript for submission.

The content of this manuscript has not been published nor submitted for publication in any other journal.

Table S1. Primers utilized in this study.

Primer name	Sequence 5'→3'	Purpose
**Xeno::pelB_Inv_fw**	aattcattaaagaggagaaattaactatgatgacaacgaagggaaacgacaagaatcctg	Cloning BVMO$_{Xeno}$::pelB_*cscA*
**Xeno::pelB_Inv-rv**	tttcatttcgattcctttatcagtgggtcaggcaggctcgacagtttccc	
**pQE-30-lin-fw**	ttagctgagcttggactcctgttgatagatccagt	Vector linearization (*cscA*$^+$)
**pQE-30-lin-rv**	catagttaatttctcctctttaatgaattctgtgtgaaattgttatc	
**cPCR-XenoInv-fw**	gaaattaactatgatgacaacgaagggaaacgacaagaatc	cPCR pQE-30:: BVMO$_{Xeno}$::pelB_*cscA*
**cPCR-XenoInv-rv**	aggtcattactggatctatcaacaggagtccaagc	
**cPCR-Xeno-fw**	gaaattaactatgatgacaacgaagggaaacgacaagaatc	cPCR pQE-30:: BVMO$_{Xeno}$ (*cscA*$^-$)
**cPCR-Xeno-rv**	attcctttatcagtgggtcaggcaggctcgac	
**Xeno-cntrl-fw**	aattcattaaagaggagaaattaactatgatgacaacgaagggaaacgacaagaatcctg	Cloning BVMO$_{Xeno}$
**Xeno-cntrl-rv**	ggatctatcaacaggagtccaagctcagctaatcaggcaggctcgacagtttccc	
**pQE-30-cntrl-lin-fw**	ttagctgagcttggactcctgttgatagatccagt	Vector linearization (*cscA*$^-$)
**pQE-30-cntrl-lin-rv**	catagttaatttctcctctttaatgaattctgtgtgaaattgttatc	
**Xeno_Inv-fw**	tgtcgagcctgcctgacccactgataaaggaatcgaaatgacgcaatctcgattgc	Cloning BVMO$_{Xeno}$_*cscA*
**Xeno_Inv-rv**	ggctgcgccccgacacccgccaacacccgctgacgcgccctgacgggcttgt	
**pQE-30-Xeno-Inv-fw**	tgccgggagcagacaagcccgtcagggcgcgtcagcgggtgttggcgggtgtc	Vector linearization (*pelB*$^-$)
**pQE-30-Xeno-Inv-rv**	gcaatcgagattgcgtcatttcgattcctttatcagtgggtcaggcaggctcgaca	

**pQE-30::Xeno-pelB_cscA**	caaacggtgctcttctacttc	Sequencing primer

Table S2. GC-FID conditions utilized in this study.

**GC-FID Parameters**		**Substrate | Product**					
		**Cyclohexanone**			**ε-caprolactone**		
**Column parameters**	Column	ZB-5, ID 342390					
	Film Thickness	0.25 µm					
	Column length	30 m					
	Inner Diameter	0.32 mm					
	Stationary phase	5 % Phenyl 95 % Dimethylpolysiloxane					
**Autosampler and Injection Port**	Injection Volume	1 µL					
	Injection Temp.	230 °C					
	Carrier Gas	N_2					
	Total Flow	21.6 mL min^{-1}			24 mL min^{-1}		
	Column Flow	0.88 mL min^{-1}			1 mL min^{-1}		
	Linear Velocity	17.6 cm s^{-1}			19.6 cm s^{-1}		
	Purge Flow	3 mL min^{-1}					
	Split Ratio	20					
**Temperature program**		**Rate [°C min^{-1}]**	**Temp. [°C]**	**Hold [min]**	**Rate [°C min^{-1}]**	**Temp. [°C]**	**Hold [min]**
		---	60	5	---	60	5
		30	310	4	10	200	3
					25	300	3
**FID**	Temperature	320 °C					
	Sampling rate	40 ms					

	H_2 flow	40 mL min^{-1}
	Air flow	400 mL min^{-1}
**Retention Times**	Cyclohexanone	9.4 min
	ε-caprolactone	14.6 min
	Cyclohexanol	9.0 min

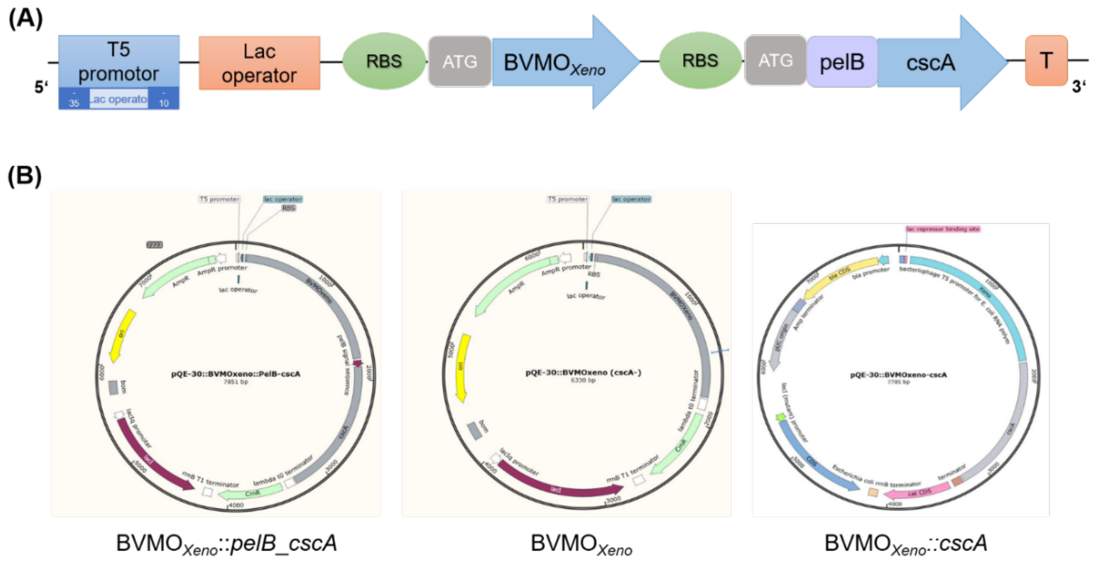

Figure S1. (A) Cloning strategy for the construction of sucrose-utilizing strains containing recombinant BVMO and invertase genes expressed either in the perisplasm (BVMO$_{Xeno}$::*pelB_cscA*) or in the cytosol (BVMO$_{Xeno}$::*cscA*). ATG- start codon; RBS- ribosome binding site; T- terminator. (B) Plasmid maps constructed for the recombinant *E. coli* strains: BVMO$_{Xeno}$::*pelB_cscA* for the expression of the target gene (BVMO$_{Xeno}$) into the cytosol and co-expression of the invertase (*pelB_cscA)* into the periplasm; BVMO$_{Xeno}$ control without additional invertase in order to show its effect; and BVMO$_{Xeno}$::*cscA* for the co-expression of both genes into the cytosol.

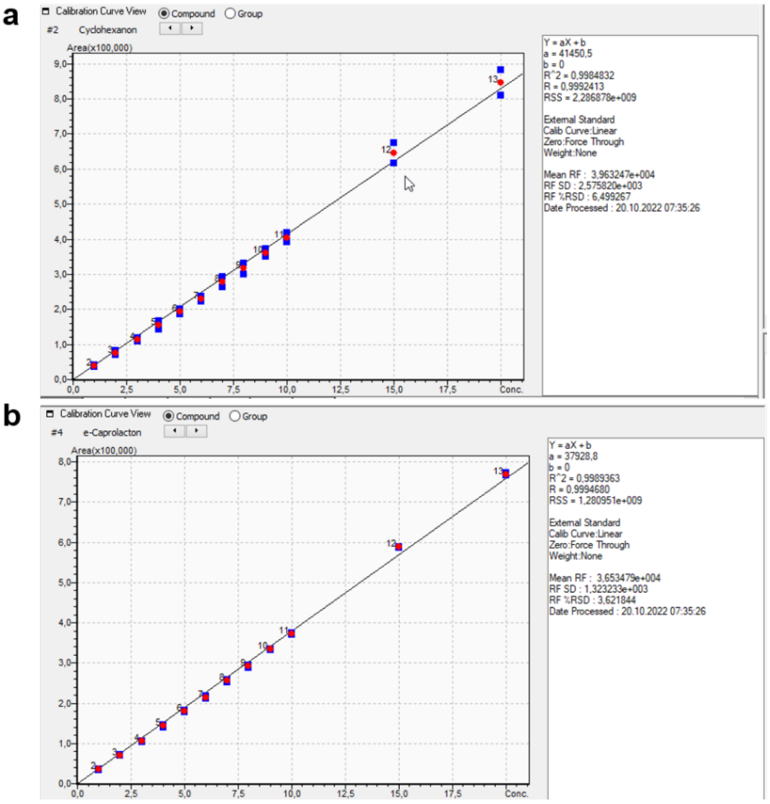

Figure S2. Calibration curves generated in LabSolutions Software for **1a** (panel **a**) and **1b** (panel **b**). In the right window of each panel, there is a corresponding equation, which was used throughout the experiments, and the R^{2}-values.

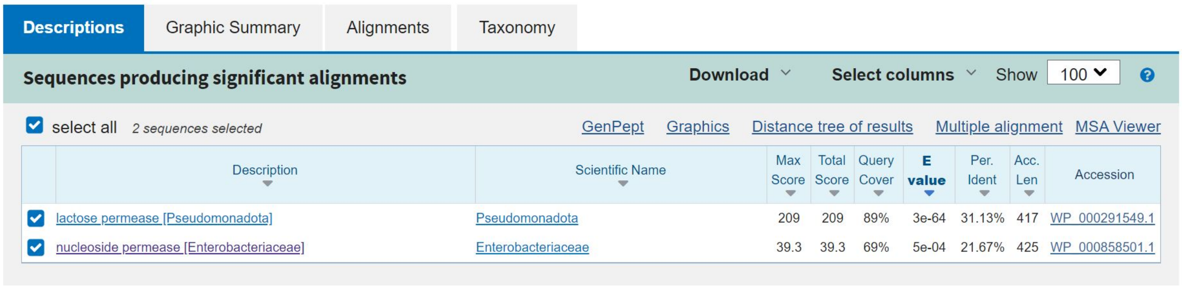

Figure S3. BLAST search of the *cscB* (accession AAC33123.1) gene sequence of *E. coli* W strain against the *E. coli* BL21 (DE3) (taxid:469008) genome. The target gene was not found and the highest homology found is with a lactose permease.

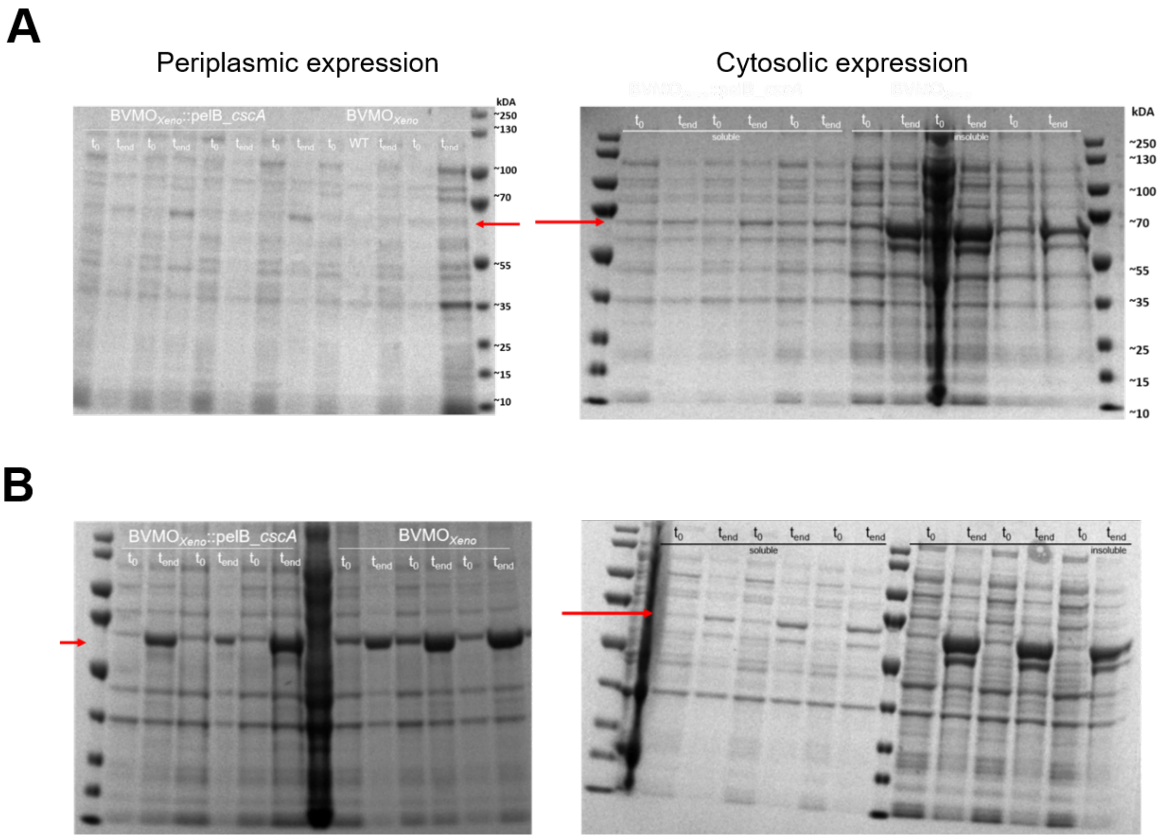

Figure S4. SDS-PAGE analyses. Both gels represent soluble fractions of three biological replicates in A) *E. coli* W ΔcscR and B) *E. coli* BL21 (DE) strains. Three strategies are shown: periplasmic invertase expression of BVMO$_{Xeno}$::pelB_*cscA* (left side) and BVMO$_{Xeno}$ (right) and cytosolic expression of BVMO$_{Xeno}$::*cscA* (far right). The respective sizes of BVMO$_{Xeno}$ and *cscA* are 62.3 and 54 kDa. Red arrow implies the expression of a BVMO.

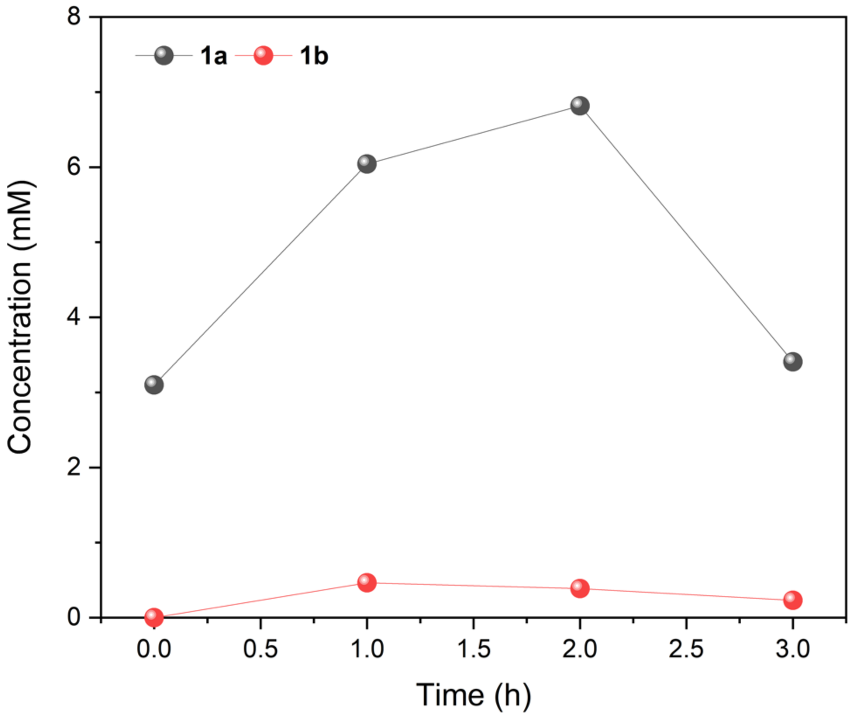

Figure S5. Biotransformation of **1a** using photosynthetically-derived sucrose from *Synechocystis* S02 mediated by *E. coli* BL21 expressing $BVMO_{Xeno}$ lacking the invertase gene.

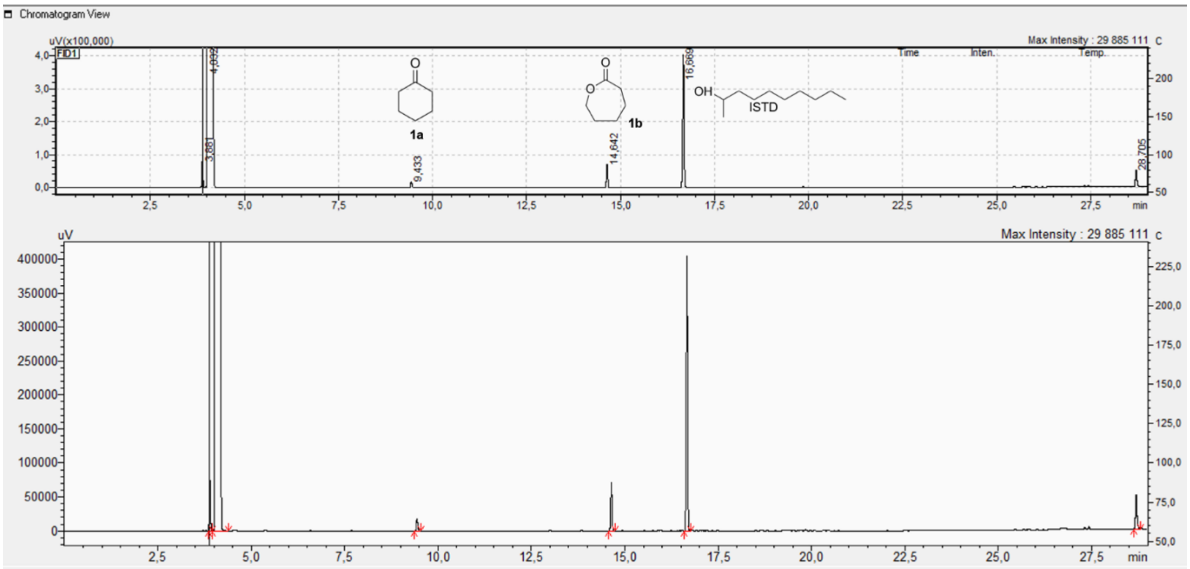

Figure S6. Sample GC-FID chromatogram during whole-cell biotransformation of **1a** mediated by *E. coli* W *ΔcsR* $BVMO_{Xeno}$::*cscA* using 1 mM sucrose after 1.5 h.
